# Genomic surveillance of SARS-CoV-2 positive passengers on flights from China to Italy, December 2022

**DOI:** 10.2807/1560-7917.ES.2023.28.2.2300008

**Published:** 2023-01-12

**Authors:** Federica Novazzi, Emanuela Giombini, Martina Rueca, Andreina Baj, Lavinia Fabeni, Angelo Genoni, Francesca Drago Ferrante, Giulia Gramigna, Cesare Ernesto Maria Gruber, Sara Boutahar, Claudia Minosse, Ornella Butera, Renee Pasciuta, Daniele Focosi, Alberto Colombo, Andrea Antinori, Enrico Girardi, Francesco Vaia, Fabrizio Maggi

**Affiliations:** 1 Laboratory of Microbiology, ASST SetteLaghi, Varese, Italy; 2 Department of Medicine and Surgery, University of Insubria, Varese, Italy; 3 National Institute for Infectious Diseases “L. Spallanzani” - IRCCS, Rome, Italy; 4 North-Western Tuscany Blood Bank, Pisa University Hospital, Pisa, Italy

**Keywords:** SARS-CoV-2, COVID-19, China, BF.7.14, BA.5.2.48, BA.5.2.49, BQ.1.1, immune escape

## Abstract

With numbers of COVID-19 cases having substantially increased at the end of 2022 in China, some countries have started or expanded testing and genomic surveillance of travellers. We report screening results in Italy in late December 2022 of 556 flight passengers in provenance from two Chinese provinces. Among these passengers, 126 (22.7%) tested SARS-CoV-2 positive. Whole genome sequencing of 61 passengers’ positive samples revealed Omicron variants, notably sub-lineages BA.5.2.48, BF.7.14 and BQ.1.1, in line with data released from China.

Following relaxation since September 2022, of measures related to ‘zero-COVID’ policies, a substantial increase in coronavirus disease (COVID-19) cases has been observed in China [1,2]. In a preprint article, the cumulative infection attack rate (i.e. the proportion of population who has been infected since 1 November) in Beijing was reported to be 43.1% (95% credible interval (CrI): 25.6–60.9) on 14 December and 75.7% (95% CrI: 60.7–84.4) on 22 December 2022 [3]. In reaction to this situation, a number of countries have recently required passengers on planes from mainland China to have a negative SARS-CoV-2 test, taken shortly pre-departure [4,5]. Moreover, some countries are testing incoming passengers from China for SARS-CoV-2 at arrival and/or are conducting/expanding genomic surveillance of travellers to monitor SARS-CoV-2 variants [4-6].

Here we report the results of screening that was conducted in Italy between 26 December and 29 December 2022 on flight passengers from China arriving at two major airports, namely Fiumicino Leonardo da Vinci airport in Rome and Malpensa airport in Milan. 

## Screening passengers for SARS-CoV-2 and variants identified

A total of 556 passengers from four flights were screened for SARS-CoV-2. Of these, 126 (22.7%) tested positive. The proportion of passengers detected with SARS-CoV-2 per flight ranged from 11 to 42% (Table). For 61 passengers (10.9% of the total 556; mean age: 49 years; 60% male; 40% females), who tested SARS-CoV-2 positive with a PCR quantification cycle (Cq) ≤ 25.0, respective samples were subjected to whole genome sequencing by using next-generation sequencing (NGS) technology in two reference laboratories. The mean Cq derived from samples of these 61 passengers was 22.9 (range: 11.1–25.0).

**Table:** Number of passengers screened and testing positive for SARS-CoV-2 among incoming flights from China, as well as characteristics of sequenced viral strains (n = 61), stratified by city of departure and arrival airport in Italy, 26–29 December 2022 (n = 556 passengers screened)

Arrival airport (town) in Italy	Date in 2022	Flight provenance: city (province) of China	Total number of passengers	Number of passengers testing SARS-CoV-2-positive^a^	Percentage of passengers testing SARS-CoV-2 positive	Number of passengers for whom a viral sequence was obtained	Number of passengers according to: virus sub-lineage	GISAID entries
Malpensa airport (Milan)	26 Dec	Nanjing (Jiangsu)	177	33	19	13	3: BA.5.2.48 4: BF.7.14 3: BQ.1.1 + ORF1a:E754K 3 NA	EPI_ISL_16343199; EPI_ISL_16343202; EPI_ISL_16343206; EPI_ISL_16343207; EPI_ISL_16343200; EPI_ISL_16343201; EPI_ISL_16343209; EPI_ISL_16343203; EPI_ISL_16343205; EPI_ISL_16343208
	26 Dec	Wenzhou (Zhejiang)	149	62	42	18	9: BA.5.2.48 5: BF.7.14 2: BQ.1.1 + ORF1a:E754K 2: NA	EPI_ISL_16350707; EPI_ISL_16350809; EPI_ISL_16350829; EPI_ISL_16352121; EPI_ISL_16352120; EPI_ISL_16350963; EPI_ISL_16350831; EPI_ISL_16352309; EPI_ISL_16352019; EPI_ISL_16350964; EPI_ISL_16352119; EPI_ISL_16352115; EPI_ISL_16352093; EPI_ISL_16352118; EPI_ISL_16352122; EPI_ISL_16352117
Fiumicino Leonardo da Vinci airport (Rome)	29 Dec	Hangzhou (Zhejiang)	46	5	11	5	3: BA.5.2.48 2: BF.7.14	EPI_ISL_16355498; EPI_ISL_16355496; EPI_ISL_16355500; EPI_ISL_16354105; EPI_ISL_16354104
	29 Dec	Wenzhou (Zhejiang)	184	26	14	25	15: BA.5.2.48 7: BF.7.14 2: BQ.1.1 + ORF1a:E754K 1: NA	EPI_ISL_16355502; EPI_ISL_16355499; EPI_ISL_16355503; EPI_ISL_16355501; EPI_ISL_16355504; EPI_ISL_16354103; EPI_ISL_16355505; EPI_ISL_16354102; EPI_ISL_16355487; EPI_ISL_16355484; EPI_ISL_16354101; EPI_ISL_16355486; EPI_ISL_16355489; EPI_ISL_16355488; EPI_ISL_16355485; EPI_ISL_16355495; EPI_ISL_16354100; EPI_ISL_16354099; EPI_ISL_16355492; EPI_ISL_16355491; EPI_ISL_16355494; EPI_ISL_16355493; EPI_ISL_16355490; EPI_ISL_16355497

NA: not assigned due to low sequence coverage; SARS-CoV-2: severe acute respiratory coronavirus 2.

^a^ According to the local regulations, SARS-CoV-2 infection was evaluated by real-time PCR and antigen detection in Malpensa airport (Milan) and Fiumicino Leonardo da Vinci airport (Rome), respectively. Antigen positive samples were then confirmed by real-time PCR.

The Table shows that three Omicron (Phylogenetic Assignment of Named Global Outbreak (Pango) lineage: B.1.1.529) sub-lineages are dominant: BA.5.2.48 (i.e. BA.5.2+T17208C) (n = 30, 49%), BF.7.14 (i.e. BF.7 + ORF7a:H47Y + S:C1243F) (n = 18, 29%), and BQ.1.1 + ORF1a:E754K (n = 7, 11%). None of these sub-lineages detected from China have spike receptor-binding domain (RBD) mutations compared with their parental lineages, and therefore likely do not pose any threat in terms of immune evasion. None of the mutations detected have been clearly associated with any changes in transmissibility or disease severity.

## Variant sub-lineage distributions according to passengers’ departure town and comparison with data from China

**Figure 1:**
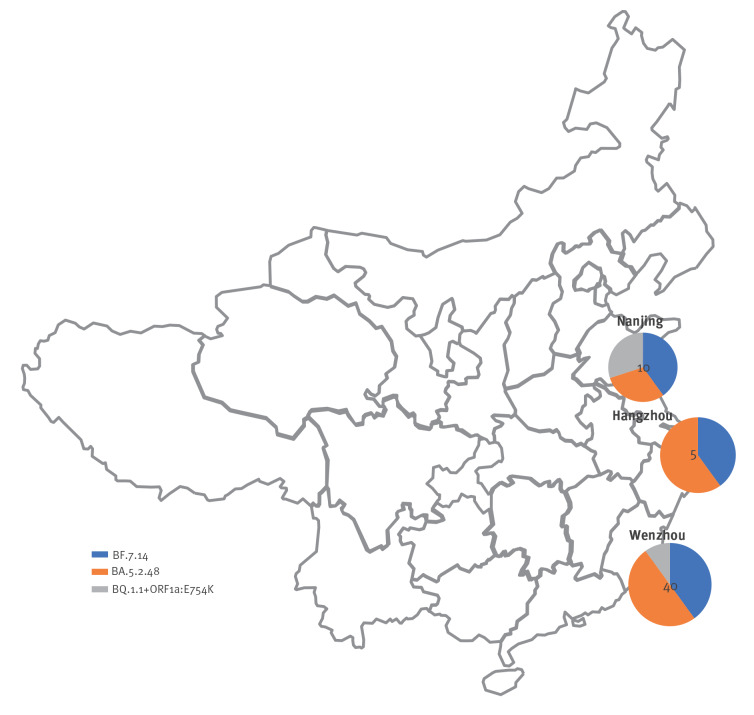
Geographic distribution of Omicron^a^ sub-lineages among passengers testing positive for SARS-CoV-2, according to the Chinese town of provenance of their flights to Italy, 26–29 December 2022 (n = 55 sequences)^a^

The flights arriving to Italy from which passengers were screened originated from three cities in East China (Figure 1) including one (Nanjing) in Jiangsu province and two (Hangzhou and Wenzhou) in Zhejiang province.

SARS-CoV-2: severe acute respiratory coronavirus 2.

^a^ Omicron (Phylogenetic Assignment of Named Global Outbreak (Pango) lineage B.1.1.529).

^b^ Of 61 passengers testing positive for SARS-CoV-2 and for whom samples were sequenced, six could not be assigned a sub-lineage due to low sequence coverage.

The disks (pie charts) are subdivided according to the proportion of each sub-lineage found. The numbers at the centres of the disks represent the total number of characterised viral sequences that were obtained through the travellers to Italy from the Chinese geographical locations in question.

On 11 January 2023 Chinese scientists deposited 708 SARS-CoV-2 sequences from China in GISAID. These were from samples from 19 November to 5 January 2023, which were not labelled as ‘imported’ in metadata.

**Figure 2:**
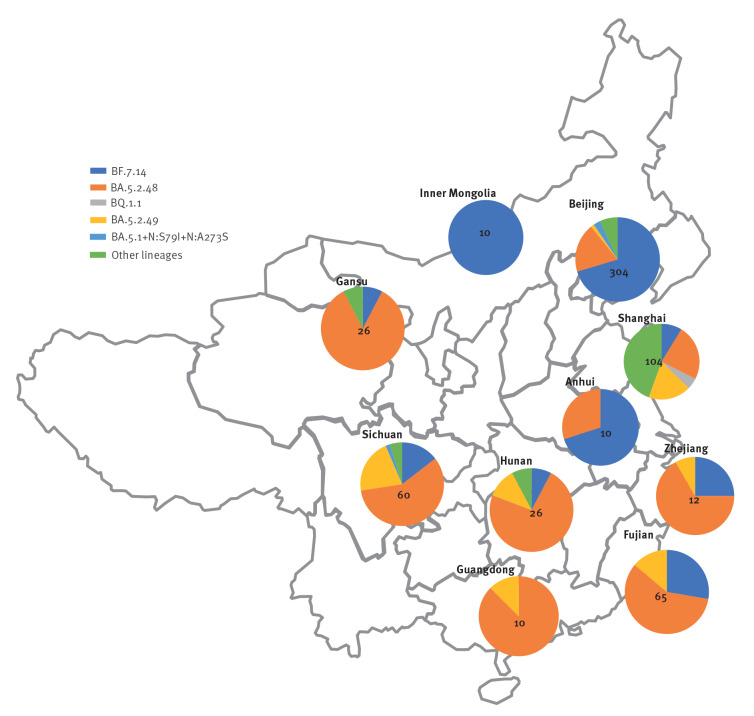
Geographic distribution by province of Omicron^a^ sub-lineages among sequences^b^ with case date after 1 September 2022 deposited from China in GISAID as of 11 January 2023 (n = 627 sequences)

^a^ Omicron (Phylogenetic Assignment of Named Global Outbreak (Pango) lineage B.1.1.529).

^b^ In the respective metadata, the deposited sequences were not labelled in as ‘imported’.

They come from 10 Chinese towns, and the distribution of sub-lineages is depicted according to the province or region that the towns belong to in Figure 2; sequences in our cohort largely overlap in type and frequency of detected sub-lineages with the GISAID sample, thereby aligning with Chinese-released data; however, data are still lacking from some parts of central and western China. While there is no reason at the moment to be concerned about novel variants [7], ongoing monitoring is strongly recommended for such an unprecedented situation.

Other lineages include BA.5.2 (n = 9), BF.21 (n = 3), BF.7 (n =1), BA.5.1 (n = 8)in Beijing, BA.2 (n = 4), BA.5 (n = 1), BA.5.1 (n = 1), BA.5.2.1 (n = 4), other BA.5.2.* (n = 34), BE.1.4.2 (n = 1), BF.7 (n = 3), BQ.1 (n = 1), BQ.1.1 (n = 4), BQ.1.1.4 (n = 1), BQ.1.23 (n = 1), CH.1.1 (n = 1) in Shanghai, BA.5.2 in Gansu (n = 2), BA.5.2 in Huanan (n = 2), BA.5 (n = 1) and BA.5.2 (n = 1) in Guangdong, and BA.5.1 (n = 1) in Sichuan.

The disks (pie charts) are subdivided according to the proportion of each sub-lineage found. The numbers at the centres of the disks represent the total number of characterised viral sequences that originated from the provinces in question.

## Discussion

In this study, we tested 556 flight passengers in provenance from China for SARS-CoV-2. Whole genome sequencing of samples respectively obtained from 61 passengers who tested positive for the virus revealed Omicron variants, notably sub-lineages BA.5.2, BF.7, and BQ.1.1 (each with additional mutations), in line with data released from China.

Among those dominant sub-lineages, BF.7.14 (i.e. BF.7 + ORF7a:H47Y + S:C1243F) deserves a special attention: BF.7, which is characterised by the S:R346T immune escape mutation, is resistant to cilgavimab + tixagevimab but instead retains sensitivity to bebtelovimab [8-10] and convalescent plasma from vaccinees [11]. So far COVID-19 waves have been driven by SARS-CoV-2 variant of concerns (VOC) associated with major changes in the RBD of the spike protein, but mutations in the S2 subunit of spike such as C1243F are unlikely to drive immune evasion. Hence, attention should be paid at the role of ORF7a:H47Y. ORF7a is an accessory cysteine-rich, zinc-binding type I transmembrane protein structurally conserved throughout the different SARS-CoV-2 VOC. It includes three separate domains: an N-terminal signal sequence (15 residues), an ectodomain with Ig-like fold (beta sandwich), a short transmembrane domain, and a C-terminal cytoplasmic tail containing a di-lysine motif (^117^KRKTE^121^) for endoplasmic reticulum (ER) localisation. Among the various interactions with host proteins, ORF7a interacts specifically with the major histocompatibility complex (MHC)-I heavy chain, acting as a molecular mimic of β_2_-microglobulin (β_2_m) to inhibit MHC-I heavy chain association with β_2_m; this slows the exit of properly assembled MHC-I molecules from the ER [12], potentially limiting cell-mediated immune responses. Zheng et al. found that F59, absent in SARS-CoV, is associated with such function [13]. ORF7a genetic sequence is also the origin for CoV2-miR-O7a, a viral miRNA-like small RNA which modulates interferon signalling via basic leucine zipper ATF-Like transcription factor 2 (BATF2) [14]. ORF7a:H47Y mutation in the ectodomain had been previously reported from Oceania and North America as early as in 2020 [15], and in the BF.5 lineage which is dominant in Japan at the end of 2022. Several other mutations, also around the F59 position, emerged recently (e.g. S44P in BA.5.11 and T61S in XAY) [16,17]. Several gain-of-function ORF7a mutations have already been associated with more severe disease presentations, e.g. A105V [18].

Inactivated whole-virus vaccines (CoronaVac from Sinovac Biotech Ltd and BBIBP-CorV from Sinopharm, Beijing, China) have been largely deployed in China, with a mean of 2.1 doses per inhabitant [19]. However, less than half of people aged 80 years and over have received three doses of the vaccine [20]. Those vaccines achieve lower risk reduction for hospitalisation [21], and lower neutralising antibodies geometric mean titres than spike-only mRNA vaccines [22]. The latter outcome could reduce the selective pressure for SARS-CoV-2 to evade anti-spike antibody response, maintaining however pressure on viral selective mechanisms to escape host cell-mediated immune response.

The BF.7 variant has been circulating in United States and Europe since August 2022 [12-14], and in Inner Mongolia since September 2022. The apparently high prevalence of BF.7.14 in China is presumably due to a founder effect: selection pressure from immune escape is much lower in China, where the contribution of previous infection-elicited immunity is less relevant than elsewhere, so it makes sense not to expect a rapid takeover by BQ.1* and XBB*. With 5 million new cases per day estimated in China, the evolution of the situation is somewhat uncertain [23].

Limitations of this study include the small sample size, the biased clinical presentation (only asymptomatic or mild patients are likely to embark on intercontinental flights and pass security checks) and the geographic restriction to the East of China.

## Conclusion

Our findings agree with sequencing data released from China and underline the relevance of genomic surveillance to detect evolution of dominant lineages in a large ecological setting. Such setting, consisting mostly of a highly vaccinated but infection-naïve population is so far unprecedented in the COVID-19 pandemic. 
